# Clinical Performance of the Elecsys HCV Duo Immunoassay Compared with a Standard-of-Care Algorithm Using Alinity Anti-HCV Screening and HCV RNA Confirmation

**DOI:** 10.3390/diagnostics16193239

**Published:** 2026-10-07

**Authors:** Jaeeun Yoo, Hye-Sun Park, JoungOk Kim, Sang-Ik Cho, Seong Hwan Jo, Seo-A Park, Min-Seok Kwon, Ae-Ran Choi, Eun-Jee Oh

**Affiliations:** 1 Department of Laboratory Medicine, Incheon St. Mary’s Hospital, College of Medicine, The Catholic University of Korea, Incheon 21431, Republic of Korea; focused@catholic.ac.kr; 2 Department of Laboratory Medicine, Seoul St. Mary’s Hospital, College of Medicine, The Catholic University of Korea, Seoul 06591, Republic of Korea; 3 Research and Development Institute for In Vitro Diagnostic Medical Devices, College of Medicine, The Catholic University of Korea, Seoul 06591, Republic of Korea; 4 Department of Medical Sciences, Graduate School, The Catholic University of Korea, Seoul 06591, Republic of Korea

**Keywords:** hepatitis C virus, HCV core antigen, anti-HCV antibody, Elecsys HCV Duo, clinical performance evaluation

## Abstract

**Background/Objectives:** The Elecsys HCV Duo immunoassay simultaneously detects HCV core antigen (HCV Ag) and anti-HCV antibodies in a single sample. We evaluated its clinical performance against a standard-of-care reference algorithm of anti-HCV antibody and HCV RNA testing. **Methods:** We retrospectively analyzed 1332 residual serum or plasma samples tested with reference HCV RNA assays and the Alinity i Anti-HCV assay. Samples were classified as reference-positive, indicating current or past HCV infection (*n* = 166; 88 RNA+/Ab+ with active infection and 78 RNA−/Ab+ with resolved or treated infection), or reference-negative (n = 1166; RNA−/Ab−), including 1000 randomly selected blood donor samples. All specimens were tested with the Elecsys HCV Duo (cobas^®^ e 801, Roche Diagnostics). Positive and negative percent agreements (PPA and NPA) were calculated against the combined reference standard. Results were further analyzed by infection status and viral load. No HCV RNA+/anti-HCV− (window-period) specimens were available. **Results:** Overall agreement was high (PPA 98.8% [164/166; 95% CI, 95.7–99.9%]; NPA 99.9% [1165/1166; 95% CI, 99.5–100.0%]). Agreement of the anti-HCV component with the Alinity i Anti-HCV assay was identical (PPA 98.8%, NPA 99.9%). The HCV Duo Ag component showed a PPA of 60.2% (95% CI, 49.2–70.5%) and NPA of 99.0% (95% CI, 98.3–99.5%) against HCV RNA detection. The Ag module was also reactive in 12 of 78 (15.4%) RNA−/Ab+ specimens, all from previously treated patients; thus, 53 of 65 (81.5%) dual Ag/Ab-reactive specimens had detectable HCV RNA. HCV Ag positivity was highest at HCV RNA ≥10^6^ IU/mL (77.4%, 41/53), compared with 32.1% (9/28) at 10^4^–10^6^ IU/mL and 42.9% (3/7) at <10^4^ IU/mL. Receiver operating characteristic analysis of the Ag component yielded an area under the curve of 0.920 (95% CI, 0.904–0.934). **Conclusions:** The Elecsys HCV Duo demonstrated high overall agreement with the reference methods, supporting its potential as a single-step screening test for HCV infection. Most, but not all, dual Ag/Ab-reactive specimens were viremic, so this result may help prioritize anti-HCV-positive individuals for linkage to care; however, the assay does not replace HCV RNA testing, which remains necessary to confirm active infection, particularly in Ag-negative or previously treated individuals. Because no window-period specimens were included, the potential of the assay for earlier detection of acute infection could not be evaluated.

## 1. Introduction

Hepatitis C virus (HCV) infection remains a major global health burden, with an estimated 57.8 million people living with chronic HCV infection worldwide and approximately 78.6% of cases remaining undiagnosed [1]. HCV is a hepatotropic, single-stranded RNA virus that frequently establishes chronic infection and can progress to liver cirrhosis and hepatocellular carcinoma [2,3]. With most acute and chronic infections being clinically silent, laboratory screening remains the cornerstone of timely detection and access to highly effective direct-acting antiviral therapies, which cure more than 90% of treated patients [2]. Global modelling and World Health Organization surveillance data similarly indicate that most people living with HCV infection remain undiagnosed or untreated [4,5]. In Korea, anti-HCV seroprevalence is approximately 0.6%, but only about one-third of anti-HCV-positive individuals are viremic, and linkage-to-care and treatment rates remain below the World Health Organization elimination targets [6].

Standard HCV testing algorithms begin with anti-HCV antibody screening, followed by HCV RNA testing to confirm active infection in antibody-reactive samples [7,8]. However, antibody-only screening has well-recognized limitations. Antibody seroconversion can be delayed for several weeks after acute infection, creating a diagnostic window during which HCV RNA is already detectable but anti-HCV is not. In addition, anti-HCV antibody reactivity persists indefinitely and cannot distinguish active infection from spontaneously cleared or successfully treated past infection, necessitating sequential RNA testing for confirmation, ideally as reflex testing on the same specimen [9,10].

Despite its role as the reference test for current infection, nucleic acid amplification testing (NAAT) is costly, technically demanding, and often centralized, with turnaround times that may exceed 24 h and limited availability in resource-constrained or non-tertiary settings. These logistical barriers can lead to missed or delayed RNA testing after an initial reactive antibody result, particularly among marginalized or hard-to-reach populations, thereby increasing loss to follow-up and delaying linkage to care. In Japanese hospital settings, for example, only 22.7–29.2% of anti-HCV-positive patients underwent confirmatory HCV RNA testing [11,12]. Accordingly, the World Health Organization now recommends simplified approaches, including reflex and point-of-care HCV RNA testing, to reduce loss to follow-up [13]. In this context, assays that directly detect HCV core antigen, particularly when combined with anti-HCV antibody in a single Ag/Ab format, have emerged as a promising alternative or adjunct to NAAT.

The Elecsys HCV Duo (Roche Diagnostics GmbH, Mannheim, Germany) is an electrochemiluminescence immunoassay that simultaneously detects HCV core antigen and anti-HCV antibodies in two parallel reaction modules from a single sample. HCV core antigen is a direct viral marker of ongoing HCV infection and a surrogate marker of viral replication. It becomes detectable in serum shortly after HCV RNA and before anti-HCV seroconversion [14,15], while the anti-HCV module preserves the diagnostic value of antibody-based screening for established infection. The simultaneous detection of both markers can therefore shorten the diagnostic window, enhance recognition of active infection among antibody-reactive individuals, and may streamline the diagnostic pathway by helping to prioritize individuals for confirmatory testing and linkage to care, although confirmation of active infection still relies on HCV RNA testing [7,8,9,10].

In this study, we evaluated the clinical performance of the Elecsys HCV Duo immunoassay against established HCV RNA and anti-HCV reference methods in a large retrospective cohort of residual serum and plasma samples drawn from clinical and blood donor populations, and we report performance separately for active infection, resolved or treated past infection, and uninfected individuals.

## 2. Materials and Methods

### 2.1. Study Design and Specimens

This retrospective performance evaluation of the Elecsys HCV Duo immunoassay (Roche Diagnostics GmbH, Mannheim, Germany) was conducted at Seoul St. Mary’s Hospital, The Catholic University of Korea, between August and November 2025. A total of 1332 anonymized residual serum or plasma specimens were enrolled. The HCV-positive group (n = 166) comprised anti-HCV-reactive specimens from outpatients of Seoul St. Mary’s Hospital (n = 83 serum) and HCV RNA-positive specimens supplied by Seoul Clinical Laboratories (n = 83 plasma). Among the 83 anti-HCV-reactive outpatient specimens, 5 were also HCV RNA-positive. The HCV-negative group (n = 1166) comprised anti-HCV-nonreactive and HCV RNA-negative specimens from inpatients of Seoul St. Mary’s Hospital (n = 166 serum, including samples with potential for cross-reactivity) and randomly selected blood donors from Hanmaum Blood Bank (n = 1000 serum). The study was approved by the Institutional Review Board of Seoul St. Mary’s Hospital (KC25DDSV0404), and the requirement for informed consent was waived because only anonymized residual specimens were used.

### 2.2. HCV Detection Assays

The Elecsys HCV Duo is an electrochemiluminescence immunoassay performed on the cobas e 801 analyzer that simultaneously detects HCV core antigen and antibodies to HCV in two parallel reaction modules, reporting results as a cutoff index (COI). Specimens were tested in accordance with the manufacturer’s instructions: results with COI ≥1.0 were interpreted as reactive at both the module level (HCV Ag, anti-HCV) and for the overall combined result. Initially reactive specimens were retested in duplicate and were considered repeatedly reactive when at least one of the duplicate measurements yielded COI ≥1.0; otherwise, the final result was classified as nonreactive.

Reference HCV RNA testing was performed using either the cobas^®^ HCV or cobas^®^ MPX assay on the cobas^®^ 6800/8800 system (Roche Molecular Systems, Pleasanton, CA, USA) or the Alinity m HCV AMP Kit on the Alinity m system (Abbott Molecular, Des Plaines, IL, USA). HCV RNA results were obtained from routine testing at each source institution: specimens from Seoul St. Mary’s Hospital were tested with the Alinity m HCV assay, HCV RNA-positive plasma specimens from Seoul Clinical Laboratories with the cobas HCV assay, and blood donor specimens from Hanmaum Blood Bank with the cobas MPX assay. The Alinity m HCV and cobas HCV assays are quantitative, whereas the cobas MPX assay is a qualitative multiplex nucleic acid test used for blood donor screening; HCV RNA viral loads were therefore derived from the two quantitative assays. Reference anti-HCV antibodies were determined with the Alinity i Anti-HCV assay on the Alinity i analyzer (Abbott GmbH, Wiesbaden, Germany). For samples with discordant anti-HCV results between the Elecsys HCV Duo and the Alinity i Anti-HCV assay, additional testing with the Elecsys Anti-HCV II assay on the cobas^®^ e 801 analyzer (Roche Diagnostics) was performed to investigate the source of disagreement.

### 2.3. Subgroup Analysis

To examine the relationship between HCV Ag detection and viral burden, HCV RNA-positive specimens were stratified into three viral load categories (<10^4^, 10^4^–10^6^, and ≥10^6^ IU/mL), and the proportion of HCV Ag-reactive results was calculated within each category.

### 2.4. Statistical Analysis

The primary analysis compared the overall Elecsys HCV Duo result with the reference methods (HCV RNA and Alinity i Anti-HCV). A sample was defined as reference-positive when HCV RNA was detected and/or anti-HCV antibodies were reactive, and as reference-negative when HCV RNA was undetectable and anti-HCV nonreactive. Because this composite reference standard identifies current or past HCV infection (i.e., HCV seropositivity and/or viremia) rather than active infection, results were additionally analyzed separately for three infection-status categories: active infection (HCV RNA detected), resolved or treated past infection (anti-HCV reactive with undetectable HCV RNA), and uninfected (anti-HCV nonreactive and HCV RNA undetectable). Among anti-HCV-positive specimens, the ability of the HCV Ag module to distinguish active from past infection was expressed as sensitivity, specificity, and the proportion of dual Ag/Ab-reactive specimens with detectable HCV RNA. Positive percent agreement (PPA) and negative percent agreement (NPA) were calculated together with two-sided exact (Clopper–Pearson) 95% confidence intervals. As secondary analyses, agreement between the Elecsys HCV Duo HCV Ag sub-result and HCV RNA and between the Elecsys HCV Duo anti-HCV sub-result and the Alinity i Anti-HCV assay were evaluated using the same approach. Receiver operating characteristic (ROC) curve analysis was performed using the continuous HCV Duo Ag COI with HCV RNA detection as the reference. All analyses were performed using R version 4.5.0 (R Foundation for Statistical Computing, Vienna, Austria).

## 3. Results

### 3.1. Overall Agreement with the Reference Methods

Of the 1332 samples tested, 166 were classified as reference-positive (HCV RNA detected and/or anti-HCV reactive on the Alinity i Anti-HCV assay, i.e., current or past infection) and 1166 as reference-negative. The Elecsys HCV Duo classified 164 of the 166 reference-positive samples as reactive and 1165 of the 1166 reference-negative samples as nonreactive, yielding a PPA of 98.80% (164/166; 95% CI, 95.72–99.85) and an NPA of 99.91% (1165/1166; 95% CI, 99.52–100.00) (Table 1). All three discordant results involved the anti-HCV module: two samples were reference-positive (RNA−/Ab+) but nonreactive on the Elecsys HCV Duo (Ag−/Ab−), and one sample was reference-negative (RNA−/Ab−) but reactive on the Elecsys HCV Duo (Ag−/Ab+) (Table 2).

### 3.2. Performance of Elecsys HCV Duo Antigen Test

Of the 1332 specimens, 88 were dual positive (HCV RNA+/anti-HCV+), 1166 were dual negative (HCV RNA−/anti-HCV−), and 78 were anti-HCV-positive with undetectable HCV RNA (HCV RNA−/anti-HCV+) by reference testing. No HCV RNA+/anti-HCV− samples were included in this study (Table 2).

When the HCV Ag module of the Elecsys HCV Duo was compared with the HCV RNA reference, 53 of 88 HCV RNA-positive samples were HCV Ag-positive, yielding a PPA of 60.23% (95% CI, 49.23–70.51); among 1244 HCV RNA-negative samples, 1232 were HCV Ag-nonreactive, yielding an NPA of 99.04% (95% CI, 98.32–99.50) (Table 3).

Among the 78 HCV RNA−/anti-HCV+ samples with viral loads below the lower limit of detection (< 12 IU/mL), 12 were Elecsys HCV Duo Ag-reactive. All 12 Ag-reactive samples were from patients with a documented history of HCV infection and antiviral treatment (3 with hepatocellular carcinoma and 9 with chronic hepatitis C). The RNA−/Ag+ samples had a median HCV Duo Ag COI of 8.56 (IQR, 2.33–17.65), which did not differ significantly from the median COI of 12.90 (IQR, 4.21–26.90) observed in the 53 RNA+/Ag+ samples (Figure S1). No samples tested Ag+/anti-HCV− on the Elecsys HCV Duo in this study.

### 3.3. Performance of the Elecsys HCV Duo Anti-HCV Test

When the anti-HCV module was compared with the Alinity i Anti-HCV assay as the reference test, 164 of 166 anti-HCV-reactive samples were classified as anti-HCV-reactive by the Elecsys HCV Duo (PPA, 98.80%; 95% CI, 95.72–99.85), and 1165 of 1166 anti-HCV-nonreactive samples were classified as anti-HCV-nonreactive (NPA, 99.91%; 95% CI, 99.52–100.00) (Table 3).

Among the three samples with discrepant anti-HCV results, two were reactive only on the Alinity i Anti-HCV assay (S/CO 3.38 and 1.06), and one was reactive only on the HCV Duo anti-HCV module (COI 1.08). All three samples remained reactive on repeat testing, and the HCV Duo anti-HCV results were concordant with those obtained using the Elecsys Anti-HCV II assay. Follow-up specimens from these three patients also tested repeatedly reactive on both the Alinity i Anti-HCV and Elecsys Anti-HCV II assays. None of the three patients had a documented history of HCV RNA positivity or prior HCV treatment.

**Figure 1:**
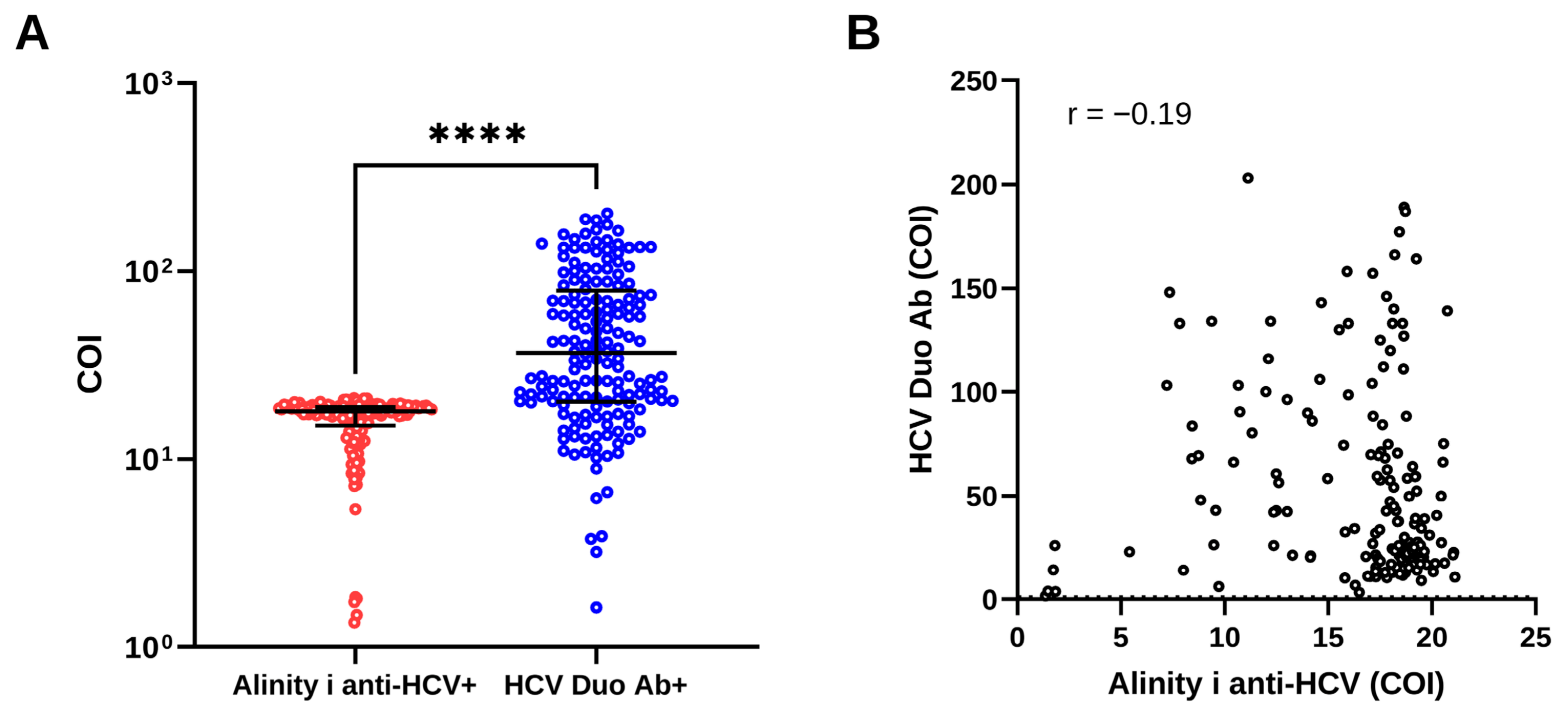
Comparison of anti-HCV signal intensity between the Elecsys HCV Duo and Alinity i assays. (**A**) Distribution of COI values for anti-HCV-reactive samples measured by the Alinity i Anti-HCV assay and by the HCV Duo Ab component. Median COI values were significantly higher with the HCV Duo Ab assay than with the Alinity i Anti-HCV assay (*p* < 0.001). (**B**) Scatter plot showing the relationship between COI values obtained with the Alinity i Anti-HCV assay and the HCV Duo Ab component for anti-HCV-reactive samples (Spearman r = −0.190). Circles represent individual samples; in (A), red and blue circles indicate the Alinity i Anti-HCV assay and the HCV Duo Ab component, respectively, and horizontal lines indicate the median and interquartile range. ****, *p* < 0.0001.

The median COI values for HCV Duo anti-HCV-negative and -positive samples were 0.023 (IQR, 0.022–0.027) and 36.400 (IQR, 20.150–76.375), respectively. The corresponding median COI values for Alinity i Anti-HCV-negative and -positive samples were 0.130 (IQR, 0.110–0.150) and 17.930 (IQR, 14.660–18.880), respectively. Anti-HCV-positive samples showed higher COI values with the HCV Duo anti-HCV assay than with the Alinity i Anti-HCV assay (*p* < 0.001, Figure 1A). COI values of the HCV Duo anti-HCV-positive samples showed only a weak correlation with those from the Alinity i Anti-HCV assay (Spearman r = −0.190, Figure 1B).

### 3.4. HCV Ag Detection by HCV RNA Viral Load

Among the 88 HCV RNA-positive samples, viral loads measured by the reference quantitative PCR assays ranged from 47 to 7.21 × 10^7^ IU/mL (median, 2.08 × 10^6^ IU/mL). HCV Ag positivity was 3/7 (42.86%; 95% CI, 9.90–81.59) at <10^4^ IU/mL, 9/28 (32.14%; 95% CI, 15.88–52.35) at 10^4^–10^6^ IU/mL, and 41/53 (77.36%; 95% CI, 63.79–87.72) at ≥10^6^ IU/mL. The detection rate was substantially higher at viral loads ≥10^6^ IU/mL than at lower viral loads.

**Figure 2:**
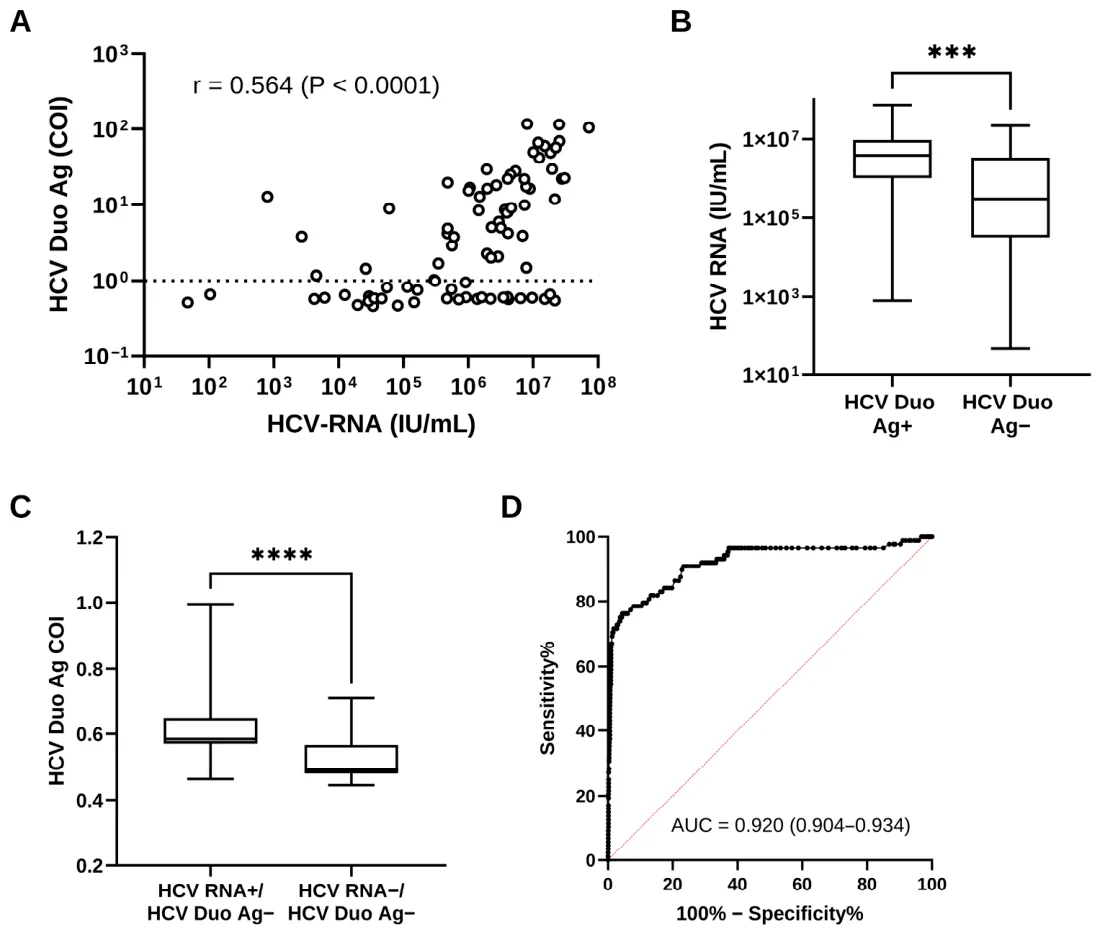
HCV Duo antigen performance according to HCV RNA level and receiver operating characteristic analysis. (**A**) Correlation between HCV Duo Ag COI and HCV RNA viral load (IU/mL) among 88 HCV RNA-positive samples (Spearman r = 0.564, *p* < 0.0001). The dotted line indicates the manufacturer’s COI cutoff of 1.0. (**B**) Box-and-whisker plots of HCV RNA viral loads in HCV Duo Ag-positive (Ag+) and Ag-negative (Ag−) HCV RNA-positive samples, showing significantly higher viral loads in Ag+ specimens. (**C**) HCV Duo Ag COI distributions in Ag-negative samples stratified by HCV RNA status, demonstrating higher COI values in RNA+/Ag− samples compared with RNA−/Ag− samples. (**D**) Receiver operating characteristic (ROC) curve for the HCV Duo Ag component for detection of HCV RNA positivity, with an area under the curve (AUC) of 0.920 (95% CI, 0.904–0.934). Circles in (A) represent individual samples. ***, *p* < 0.001; ****, *p* < 0.0001.

HCV RNA levels (IU/mL) showed a moderate positive correlation with HCV Ag levels measured by the HCV Duo Ag assay (Spearman r = 0.564; 95% CI, 0.397–0.695) (Figure 2A). Viral loads were higher in the 53 RNA+/Ag+ samples than in the 35 RNA+/Ag− samples (median, 3.88 × 10^6^ IU/mL [interquartile range (IQR), 1.03 × 10^6^–9.50 × 10^6^] vs. 3.06 × 10^5^ IU/mL [IQR, 3.14 × 10^4^–3.46 × 10^5^]; *p* < 0.001) (Figure 2B). Of the 1267 samples that were HCV Duo Ag-negative, 35 (2.8%) tested HCV RNA-positive. The 35 Ag−/RNA+ samples had a median HCV Duo Ag COI of 0.585 (IQR, 0.571–0.649), which was higher than the median COI of 0.493 (IQR, 0.481–0.567) observed in the 1232 Ag−/RNA− samples (Figure 2C). Receiver operating characteristic (ROC) curve analysis for the HCV Duo Ag component yielded an area under the curve (AUC) of 0.920 (95% CI, 0.904–0.934) (Figure 2D). At the manufacturer’s cutoff (COI 1.0), the corresponding sensitivity and specificity were 60.23% and 99.04%, respectively.

### 3.5. Performance According to HCV Infection Status

When results were analyzed separately by infection status (Table 4), the Elecsys HCV Duo was reactive in all 88 specimens with active infection (100.00%; 95% CI, 95.89–100.00), in 76 of 78 specimens with resolved or treated past infection (97.44%; 95% CI, 91.04–99.69), and in 1 of 1166 uninfected specimens (NPA, 99.91%; 95% CI, 99.52–100.00). The HCV Ag module was reactive in 53 of 88 (60.23%) specimens with active infection, in 12 of 78 (15.38%; 95% CI, 8.21–25.33) specimens with past infection, and in none of the 1166 uninfected specimens (NPA, 100.00%; 95% CI, 99.68–100.00). Thus, all 12 HCV Ag-reactive, RNA-negative results occurred in individuals with past infection. Among the 166 anti-HCV-positive specimens, the Ag module distinguished active from past infection with a sensitivity of 60.23% (53/88) and a specificity of 84.62% (66/78; 95% CI, 74.67–91.79). Of the 65 specimens reactive on both modules, 53 (81.54%; 95% CI, 69.97–90.08) had detectable HCV RNA, whereas 35 of the 99 anti-HCV-positive specimens with an Ag−/anti-HCV+ result (35.35%; 95% CI, 26.01–45.60) were HCV RNA-positive.

## 4. Discussion

In this clinical performance evaluation of the Elecsys HCV Duo immunoassay using 1332 residual serum or plasma samples, the assay demonstrated high overall agreement with the reference methods (PPA, 98.80%; 95% CI, 95.72–99.85; NPA, 99.91%; 95% CI, 99.52–100.00). At the module level, the anti-HCV sub-result agreed closely with the Alinity i Anti-HCV reference (PPA, 98.80%; NPA, 99.91%), whereas the HCV Ag sub-result identified 53 of 88 HCV RNA-positive samples (PPA, 60.23%; NPA, 99.04%), with detection notably higher at viral loads ≥10^6^ IU/mL.

Our findings are broadly consistent with previous evaluations of the Elecsys HCV Duo. A U.S. study of 1341 clinical specimens reported PPA/NPA values of 95.6%/99.1% for the anti-HCV module versus a third-generation anti-HCV assay and 73.5%/99.8% for the HCV Ag module versus nucleic acid amplification testing, while a Thai cohort of 769 samples showed 100% sensitivity of the anti-HCV module in anti-HCV-reactive/RNA-positive cases and 70.6% sensitivity of the HCV Ag module for active infection [16,17]. Similarly, a Japanese evaluation that used the same anti-HCV comparator as our study reported complete agreement (1335/1335) between the HCV Duo anti-HCV module and the Alinity i Anti-HCV assay [18]. Taken together with these data, our results support the robustness of the anti-HCV component and confirm that the overall performance of the Elecsys HCV Duo is primarily constrained by the analytical sensitivity of the antigen module.

Three samples produced discrepant anti-HCV results between the Elecsys HCV Duo and the reference algorithm. Two samples were classified as reactive only by the Alinity i Anti-HCV assay (S/CO 3.38 and 1.06) but were nonreactive on the Elecsys HCV Duo and Elecsys Anti-HCV II (Elecsys Anti-HCV II COI 0.04 and 0.04), whereas one sample was nonreactive by Alinity i Anti-HCV (S/CO 0.37) but reactive on both the HCV Duo anti-HCV module and Elecsys Anti-HCV II (HCV Duo COI 1.08; Elecsys Anti-HCV II COI 3.23). All three specimens retained the same reactivity pattern on repeat testing, and follow-up samples from these patients also remained repeatedly reactive on the same assays, without any documented history of HCV RNA positivity or prior antiviral treatment. These findings most likely reflect borderline or assay-specific low-level seroreactivity, with either the Alinity i or HCV Duo anti-HCV assay representing the source of disagreement in this small subset rather than true differences in underlying infection status. These results suggest the importance of interpreting low-level reactive anti-HCV results in conjunction with confirmatory RNA testing and clinical history, particularly when discordant findings arise between assay platforms. The anti-HCV component of the Elecsys HCV Duo in our study showed excellent concordance with the Alinity i anti-HCV assay, with a PPA of 98.80% and an NPA of 99.91% when Alinity i Anti-HCV was used as the reference. The marked separation in COI distributions between negative and positive samples on the HCV Duo anti-HCV assay, together with significantly higher COI values compared with the Alinity i Anti-HCV assay, suggests that the Elecsys platform may generate stronger signal intensities in true-positive specimens while maintaining low background reactivity in negatives. Although COI values between the two assays correlated only weakly, this is not unexpected given differences in antigen composition, assay format, and dynamic range—in particular, Alinity i S/CO values of reactive samples were compressed near the upper end of the assay’s reporting range (median 17.9)—and does not detract from the high qualitative agreement observed.

The HCV Ag module showed substantially lower sensitivity than HCV RNA testing, with a PPA of 60.23% (53/88). Similar reductions have been reported in other Elecsys HCV Duo evaluations, with HCV Ag sensitivities of 73.5% in a U.S. cohort, 70.6% in a Thai cohort, 72.2% (13/18) and 55.0% in two Japanese cohorts, and 63.2% in a South African cohort [16,17,18,19,20]. Sensitivities of 62.8% and 78% have also been reported in other Japanese cohorts [12,21], and in a French cohort the Ag module missed 95.5% of infections with viral loads of ≤5 log10 IU/mL [22]. In the Japanese evaluation by Sugiura et al., Ag-negative results predominated among specimens with HCV RNA below approximately 5.9 log IU/mL, and the authors likewise concluded that HCV Ag alone cannot replace HCV RNA testing [18]. This pattern reflects the inherent analytical sensitivity of HCV core antigen detection, whose lower limit roughly corresponds to 1000 IU/mL of HCV RNA; close agreement between core antigen and HCV RNA levels is generally observed only above approximately 3000 IU/mL, as shown in prior quantitative comparisons [23,24,25,26]. Assays with higher analytical sensitivity may narrow this gap: in a Japanese cohort, an iTACT-based core antigen assay detected 91.0% of HCV RNA-positive samples, compared with 72.9% for the HCV Duo Ag module [27]. HCV Ag detection may also depend on genotype: in the South African cohort, the HCV Duo Ag module detected 69.6% (95% CI, 59.5–79.7) of genotype 1 infections but only 43.2% (95% CI, 28.7–57.7) of non-genotype 1 infections [20]. Because genotypes 1b and 2 predominate in Korea [8], the genotype composition of our cohort may have influenced the observed Ag PPA, although genotype data were not available to examine this directly.

The Elecsys HCV Duo antigen component was reactive in 12 of 78 (15.4%) anti-HCV-positive, RNA-negative specimens, all from patients with previously treated infection. Because residual specimen volume was exhausted, these specimens could not be examined by neutralization or an alternative core antigen assay, and the nature of this reactivity therefore remains uncertain. Possible explanations include nonspecific reactivity, persistence of circulating core antigen or antigen–antibody complexes after viral clearance, and intermittent viremia below the detection limit of the RNA assays. The median Ag COI of these specimens (8.56) was comparable to that of RNA-positive, Ag-reactive specimens, which argues against low-level signal near the cutoff as the sole explanation. Persistent Ag reactivity with undetectable HCV RNA during direct-acting antiviral therapy has been described with this assay [18], whereas no Ag reactivity was observed among 50 antibody-positive, RNA-negative individuals in the South African cohort [20], suggesting that the frequency of this finding may depend on the population studied. Regardless of its mechanism, this finding shows that dual Ag/Ab reactivity did not invariably indicate detectable HCV RNA in our cohort, particularly in individuals with a history of HCV infection or antiviral treatment.

The viral load dependence of HCV Ag detection was clearly apparent in our data. Among the 88 HCV RNA-positive samples (range, 47 to 7.21 × 10^7^ IU/mL; median, 2.08 × 10^6^ IU/mL), HCV Ag positivity was 42.86% (3/7) at <10^4^ IU/mL, 32.14% (9/28) at 10^4^–10^6^ IU/mL, and 77.36% (41/53) at ≥10^6^ IU/mL (the small number of samples below 10^4^ IU/mL precludes reliable estimation in this tier). A similar stepwise increase was reported in a Japanese cohort, where HCV Ag detection rose from 20.5% at ≤10^4^ IU/mL to 33.3% at 10^4^–3 × 10^5^ IU/mL and 87.3% at ≥4 × 10^5^ IU/mL [19]. In both studies, Ag detection improved sharply with increasing viral load but remained incomplete even in the highest tier, with 13–23% of HCV RNA-positive samples not detected. Consequently, a negative HCV Ag result on the Elecsys HCV Duo cannot reliably exclude active HCV infection, particularly at low or moderate viral loads. Conversely, 53 of 65 (81.5%) dual Ag/Ab-reactive specimens had detectable HCV RNA (Table 4). In other cohorts, all dual-reactive specimens had detectable HCV RNA [22,28]; the lower proportion in our study most likely reflects the inclusion of many previously treated patients among the anti-HCV-positive specimens. Consistent with the current recommendations [7,8,9,10], we therefore suggest that a dual-reactive result be regarded as indicating probable active infection that warrants prompt referral and linkage to care, while HCV RNA testing is still performed to confirm viremia, particularly in individuals with a history of HCV infection or antiviral treatment.

The ROC analysis should be interpreted with caution. The AUC of 0.920 reflects the ability of the continuous Ag COI to rank HCV RNA-positive above HCV RNA-negative specimens across all possible thresholds, indicating good overall discrimination; however, it does not describe performance at the manufacturer’s cutoff, at which sensitivity was only 60.23%. Moreover, most RNA-negative specimens in our cohort were from uninfected individuals with very low COI values, which favors a high AUC. Clinically, the ROC result mainly supports the observation that RNA-positive specimens tend to show higher, albeit subthreshold, Ag signals than RNA-negative specimens (Figure 2C), suggesting that a lower cutoff or a borderline COI zone for repeat or reflex RNA testing could be explored. Any such threshold would require prospective validation, and in routine use, the HCV Duo should be interpreted at the manufacturer’s qualitative cutoff.

The simultaneous detection of HCV core antigen and anti-HCV antibodies by the Elecsys HCV Duo offers a practical advantage over conventional antibody-only HCV screening: the assay has the potential to detect active infection during the seronegative window period, before anti-HCV antibodies develop. In a U.S. evaluation, the Elecsys HCV Duo identified HCV in 5 of 7 (71.4%) antibody-negative, HCV RNA-positive samples that would otherwise have been missed by traditional algorithmic testing [16], and in a multicenter seroconversion panel study, it detected infection 2.2–21.9 days earlier than most comparator immunoassays [29]. Because no HCV RNA-positive/anti-HCV-negative specimens were included in our cohort, this advantage could not be evaluated in the present study. These features may be particularly relevant for screening immunocompromised patients with attenuated antibody responses, including those with HIV coinfection, in whom comparable diagnostic accuracy has been demonstrated [30], and for blood donor screening, where early detection of acute infection is critical. A combined Ab/Ag approach may therefore facilitate more rapid pathways to clinical care for HCV infection; however, the assay should be regarded as a complement to, rather than a replacement for, HCV RNA testing, which remains necessary to confirm active infection, particularly in anti-HCV-positive/Ag-negative specimens and in previously treated individuals.

This study has several limitations. The retrospective design, based on anonymized residual specimens tested at a single tertiary center, may limit the generalizability of our findings to other settings. No HCV RNA-positive/anti-HCV-negative (window-period) specimens were available, so the potential of the antigen module to shorten the diagnostic window could not be assessed directly, and our findings apply to individuals with established anti-HCV seropositivity. All HCV RNA-positive specimens from the external laboratory were plasma whereas all other specimens were serum, so specimen matrix was confounded with specimen source. The 12 HCV RNA-negative/HCV Ag-reactive specimens were not further characterized by neutralization or an alternative core antigen assay, so the cause of their reactivity remains unresolved. HCV genotype data were not available, precluding assessment of potential genotype-specific differences in core antigen detection, which have been reported previously [20]. In addition, the HCV RNA reference comprised three different molecular assays across source institutions (cobas HCV, cobas MPX, and Alinity m HCV): 83 of the 88 HCV RNA-positive specimens were quantified with the cobas HCV assay and the remaining 5 with the Alinity m HCV assay, all RNA-negative clinical specimens were tested with the Alinity m HCV assay, and donor specimens were screened with the qualitative cobas MPX assay. All three are automated real-time PCR assays with high analytical sensitivity, so misclassification of qualitative RNA status is unlikely to have materially affected the reference categories; however, differences in calibration, quantification range, and detection limits between platforms may have introduced some heterogeneity in viral load measurements [31], which could have affected the assignment of specimens near the boundaries of the viral load categories.

Several aspects of the Elecsys HCV Duo merit further investigation. Performance of the assay during early acute HCV infection should be confirmed using seroconversion panels, which would directly quantify the diagnostic window reduction relative to antibody-only screening. Because genotype-dependent differences in HCV Ag sensitivity have been reported [20], genotype-specific evaluation in Korean populations, in which genotypes 1b and 2 predominate, is also warranted. The clinical significance of Ag reactivity in previously treated, RNA-negative individuals should be clarified by follow-up RNA testing and neutralization assays. Multicenter validation across diverse clinical settings, prospective evaluation of real-world workflow benefits, and cost-effectiveness analysis comparing single-step Elecsys HCV Duo screening with conventional sequential algorithms will help to define its optimal role in routine and donor screening practice.

In conclusion, the Elecsys HCV Duo immunoassay demonstrated high agreement with established HCV RNA and anti-HCV reference assays, supporting its potential as a single-step screening test for HCV infection. Most dual Ag/Ab-reactive specimens were viremic, so this result may help prioritize anti-HCV-positive individuals for prompt linkage to care, but it did not invariably indicate detectable HCV RNA, particularly in previously treated patients. The reduced sensitivity of the HCV Ag module at low viral loads, however, indicates that the assay cannot replace HCV RNA testing, which remains necessary to confirm active infection, particularly when infection is clinically suspected or when an anti-HCV-positive/Ag-negative result is obtained. Because no window-period specimens were included, the potential of the assay for earlier detection of acute HCV infection remains to be confirmed.

## Figures and Tables

**Table 1:** Agreement of the Elecsys HCV Duo with the reference methods.

HCV Duo Result	Reference-Positive *	Reference-Negative *	Total
Reactive	164	1	165
Non-reactive	2	1165	1167
Total	166	1166	1332
Metric	Value	n/N	95% CI
Positive percent agreement (PPA)	98.80%	164/166	95.72–99.85
Negative percent agreement (NPA)	99.91%	1165/1166	99.52–100.00

* Reference methods: HCV RNA detected and/or anti-HCV reactive on the Alinity i Anti-HCV assay. Reference-positive specimens therefore include both active (HCV RNA-positive) and resolved or treated (HCV RNA-negative/anti-HCV-positive) infections. Abbreviations: CI, confidence interval.

**Table 2:** Distribution of Elecsys HCV Duo antigen/antibody results according to reference HCV RNA/antibody categories.

	Reference HCV Results			
Elecsys HCV Duo result, n (%)	HCV RNA+/ anti-HCV+	HCV RNA+/ anti-HCV−	HCV RNA−/ anti-HCV+	HCV RNA−/ anti-HCV−
	n = 88	n = 0	n = 78	n = 1166
Elecsys HCV Duo +	88 (100.0)	0 (0.0)	76 (97.4)	1 (0.1)
HCV Ag+/anti-HCV+	53 (60.2)	0 (0.0)	12 (15.4)	0 (0.0)
HCV Ag+/anti-HCV−	0 (0.0)	0 (0.0)	0 (0.0)	0 (0.0)
HCV Ag−/anti-HCV+	35 (39.8)	0 (0.0)	64 (82.1)	1 (0.1) **
HCV Ag−/anti-HCV−	0 (0.0)	0 (0.0)	2 (2.6) *	1165 (99.9)

Values are n (%). * Anti-HCV reactive on the Alinity i Anti-HCV assay but nonreactive on the Elecsys HCV Duo (potential Alinity i false positive or HCV Duo false negative); ** anti-HCV nonreactive on the Alinity i Anti-HCV assay but reactive on the Elecsys HCV Duo (potential Alinity i false negative or HCV Duo false positive).

**Table 3:** Module-level performance of the Elecsys HCV Duo.

Module	Reference Assay	PPA, % (n/N)	95% CI	NPA, % (n/N)	95% CI
HCV Ag	HCV RNA	60.23 (53/88)	49.23–70.51	99.04 (1232/1244)	98.32–99.50
Anti-HCV	Alinity i Anti-HCV	98.80 (164/166)	95.72–99.85	99.91 (1165/1166)	99.52–100.00

Abbreviations: PPA, positive percent agreement; NPA, negative percent agreement; CI, confidence interval.

**Table 4:** Elecsys HCV Duo results according to HCV infection status.

Infection Status (Reference)	n	HCV Duo-Reactive	HCV Ag-Reactive	Anti-HCV-Reactive
Active infection (HCV RNA+/anti-HCV+)	88	88 (100.0) [95.9–100.0]	53 (60.2) [49.2–70.5]	88 (100.0) [95.9–100.0]
Resolved or treated past infection (HCV RNA−/anti-HCV+)	78	76 (97.4) [91.0–99.7]	12 (15.4) [8.2–25.3]	76 (97.4) [91.0–99.7]
Uninfected (HCV RNA−/anti-HCV−)	1166	1 (0.1) [0.0–0.5]	0 (0.0) [0.0–0.3]	1 (0.1) [0.0–0.5]

Values are n (%) [exact 95% CI]. Active infection, HCV RNA detected; resolved or treated past infection, anti-HCV reactive on the Alinity i Anti-HCV assay with undetectable HCV RNA; uninfected, anti-HCV nonreactive with undetectable HCV RNA. No HCV RNA+/anti-HCV− specimens were available. Among anti-HCV-positive specimens, 53 of 65 (81.5%) dual Ag/Ab-reactive specimens and 35 of 99 (35.4%) Ag−/anti-HCV+ specimens had detectable HCV RNA.

## Data Availability

The data presented in this study are available on request from the corresponding author due to institutional restrictions on the sharing of patient-derived laboratory data.

## Supplementary Materials

The following supporting information can be downloaded at: https://www.mdpi.com/article/10.3390/diagnostics16193239/s1. Figure S1: Comparison of HCV Duo antigen COI values in Ag-reactive samples with and without detectable HCV RNA. Box-and-whisker plots showing HCV Duo Ag COI distributions in HCV RNA-positive/HCV Duo Ag-positive and HCV RNA-negative/HCV Duo Ag-positive specimens. Median COI values did not differ significantly between the two groups (ns).
