# Supplementary material for: Clinical Performance of the Elecsys HCV Duo Immunoassay Compared with a Standard-of-Care Algorithm Using Alinity Anti-HCV Screening and HCV RNA Confirmation
Source: Diagnostics (Basel). 2026 Oct 7;16(19):3239. doi: 10.3390/diagnostics16193239 (PMC13664362; doi:10.3390/diagnostics16193239)
Supplement: Supplementary file 1 [file diagnostics-16-03239-s001.zip › diagnostics-4597216-supplementary.pdf]

Supplementary Materials

Clinical Performance of the Elecsys HCV Duo Immunoassay Compared with a Standard-of-Care Algorithm Using Alinity Anti-HCV Screening and HCV RNA Confirmation

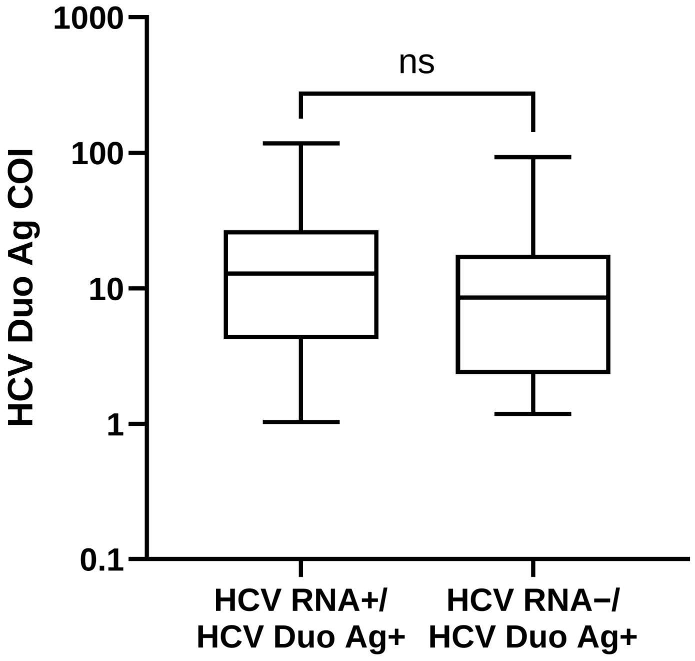

Figure S1. Comparison of HCV Duo antigen COI values in Ag-reactive samples with and without detectable HCV RNA. Box-and-whisker plots showing HCV Duo Ag COI distributions in HCV RNA-positive/HCV Duo Ag-positive and HCV RNA-negative/HCV Duo Ag-positive specimens. Median COI values did not differ significantly between the two groups (ns).
